# Review of current treatment modalities and clinical outcome of giant saccular aneurysms of the basilar apex

**DOI:** 10.1016/j.bas.2024.103333

**Published:** 2024-09-07

**Authors:** Andreas Theofanopoulos, Lucas Troude, Milad Neyazi, Sajjad Muhammad

**Affiliations:** a Department of Neurosurgery, University Hospital of Patras, Patras, Greece; b Department of Neurosurgery, Medical Faculty and University Hospital Düsseldorf, Heinrich-Heine-Universität Düsseldorf, Düsseldorf, Germany; c Department of Neurosurgery, North University Hospital Marseille, APHM-AMU, Marseille, France; d Department of Neurosurgery, University of Helsinki and Helsinki University Hospital, Helsinki, Finland

**Keywords:** Giant basilar aneurysm, Endovascular treatment, Saccular aneurysm, Surgical treatment, Outcome

## Abstract

**Introduction:**

Giant aneurysms of the basilar apex represent formidable challenges as the high rupture rate of untreated lesions must be balanced against the technical complexity and potential morbidity of intervention.

**Research question:**

Review of treatment modalities and outcomes of patients harboring giant (>2.5 cm) basilar apex saccular aneurysms, in an effort to refine treatment decision-making.

**Material and methods:**

A systematic literature review through the PubMed and Scopus databases was performed according to the PRISMA guidelines to identify cases of giant basilar apex saccular aneurysms treated either microsurgically or endovascularly. Patients’ demographics, aneurysm size, preoperative and postoperative neurologic status, angiographic and clinical outcomes as well as follow-up information were obtained.

**Results:**

Data from 32 studies fulfilling the inclusion criteria, including 49 patients (32 treated surgically and 17 endovascularly) was obtained. Mean patient age at presentation was 51.69 years, with a male-to-female ratio of 1:2. Mean maximum aneurysm diameter was 30.57 mm. A favorable outcome (mRS 0–2) was reported on 70.6% of endovascular and 56.3% of open surgical cases. Complete aneurysm occlusion was achieved in 55.6% of the open and 23.5% of the endovascular cases. Death rate was 33% for endovascular and 15.6% for open cases; the higher mortality of endovascular treatment is mainly attributed to the mass effect from continued brainstem compression after treatment.

**Discussion and conclusion:**

Higher rates of complete occlusion but higher morbidity are associated with microsurgery compared to endovascular modalities. Severe, clinically apparent brainstem mass effect may require decompression associated with microsurgery, when technically feasible.

## Highlights

• Giant (>2.5 cm diameter) saccular aneurysms of the basilar apex have malignant clinical courses and thus require treatment.
• Treatment requires complex endovascular (coiling, stenting) and microsurgical (clipping, trapping, bypass) techniques.
• Endovascular (usually first-line) treatment is associated with lower morbidity but higher recurrence rates.
• Microsurgery shows increased morbidity but higher complete occlusion rates.
• Brainstem mass effect may necessitate microsurgery for decompression.

## 1 Introduction

The overall prevalence of unruptured saccular intracranial aneurysms is estimated to be about 3% of the general population (Vlak et al., 2011). Giant intracranial aneurysms are defined by the Cooperative Study of Intracranial Aneurysms and Subarachnoid Hemorrhage as lesions with a maximum diameter greater than 25 mm (Choi and David, 2003). These lesions constitute 2–5% of all aneurysms (Locksley, 1966).

Giant aneurysms of the posterior circulation are associated with poor natural history, with a reported 50% risk of rupture within 5 years in the ISUIA trial (Wiebers, 2003a). Aneurysms of that caliber can also present with symptoms related to mass effect on the neurovascular structures and brainstem. Furthermore, due to the potentially slower blood flow and subsequent thrombosis in parts of the aneurysm lumen, clinical presentation can be in the form of transient ischemic attacks or infarction due to thromboembolism.

The poor prognosis for patients with untreated GBA argues for a treatment, the goal of which should be the safe and complete obliteration of the aneurysm. Microsurgery in this region is highly complex due to the narrow and deep space in which delicate maneuvers need to be undertaken and requires extensive experience and skill. However, satisfactory results reported in the literature point to the feasibility of this approach modality (Lawton, 2002a). In the current era, endovascular treatment (either by simple coiling or utilizing adjuncts as stents and flow-diverters) has been established as a preferred treatment for aneurysms of the posterior circulation, bypassing the morbidity of the traditional surgical approaches (Wiebers, 2003b; Ge et al., 2022; van Rooij and Sluzewski, 2009). However, in cases of giant aneurysms, endovascular treatment is technically challenging and associated with high morbidity. Furthermore, there is evidence of a greater recurrence rate (Da Ros et al., 2017; van Rooij and Sluzewski, 2007).

With no clearly defined guidelines, choice of treatment modality is at the discretion of the treating physicians and is likely to be strongly influenced by the treating centers’ relative expertise with either technique (Tjahjadi et al., 2018). In an effort to shed light on this crucial issue, we performed a systematic review of the literature to compare clinical and radiological results in aneurysms of the basilar apex treated either surgically or endovascularly.

## 2 Methods

The Preferred Reporting Items for Systematic Reviews and Meta-Analyses (PRISMA) Guidelines were used as a template for the methodology.

### 2.1 Search strategy

A comprehensive literature search was conducted through the PubMed and Scopus databases in January 2024, using the following terms: (aneurysm) AND (giant) AND (basilar).

### 2.2 Inclusion and exclusion criteria

Descriptive and observational studies including case-control, longitudinal cohorts, cross-sectional studies, retrospective studies, review articles, editorials, commentaries, case series and case reports reporting outcomes of treatment of giant aneurysms in the basilar tip were included. Video articles were excluded. We excluded aneurysms' location other than the basilar tip from our systematic review. Aneurysms with morphology described as fusiform were also excluded. Studies not specifying treatment strategy, aneurysms’ locations or patient outcomes were excluded. Studies reporting aneurysm size less than 25 mm or not clearly defining the size of giant aneurysms as >25 mm were also excluded. Finally, articles with full text in languages other than English were excluded.

### 2.3 Study selection

The duplicates from the initial database search were removed utilizing the Automated Systematic Search Deduplicator (ASySD) application and a preliminary screening based on Title and Abstract was performed. Consequently, full-text screening was performed based on the predefined inclusion and exclusion criteria. Any conflicts were discussed and resolved by the senior author. The final included articles were reviewed and approved by all authors.

### 2.4 Data extraction

The age, sex, aneurysms' maximal diameter, presenting complaints, treatment modality, perioperative complications, completeness of occlusion, preoperative and postoperative neurologic status at latest reported follow-up were tabulated and reviewed. The modified Rankin Scale (mRS) was used as a measure of clinical outcome, with mRS scores of 0–2 denoting a ‘Good’ outcome and mRS 3–6 denoting a ‘Poor’ outcome for the subsequent analysis. In studies describing the patients' clinical status without specifying an mRS score, the score was calculated by the authors based on the clinical characteristics provided. In studies that reported clinical status of individual patients as a range of GOS or mRS scores (e.g ‘Good’ outcome meaning GOS 4–5 or ‘Good’ grade SAH meaning Hunt Hess scores 1–3), the respective mean of the range was used for each patient; those patients whose preoperative status was referred just as unruptured, were regarded as mRS 1 (Nanda et al., 2014a). For studies providing the clinical outcome by measure of Glasgow Outcome Scale (GOS), the GOS score was converted to the corresponding mRS score based on the recommendations by Gaastra et al. (2022)

### 2.5 Statistical analysis

Comparison of variables was performed using the Chi-square (X^2^) test; Fisher's exact test was used instead if the expected frequency of at least an observation was less than 5. Missing observations were left blank during analysis. Statistical analysis was performed using IBM SPSS Statistics Version 28.0.1.1.

## 3 Results

**Fig. 1:**
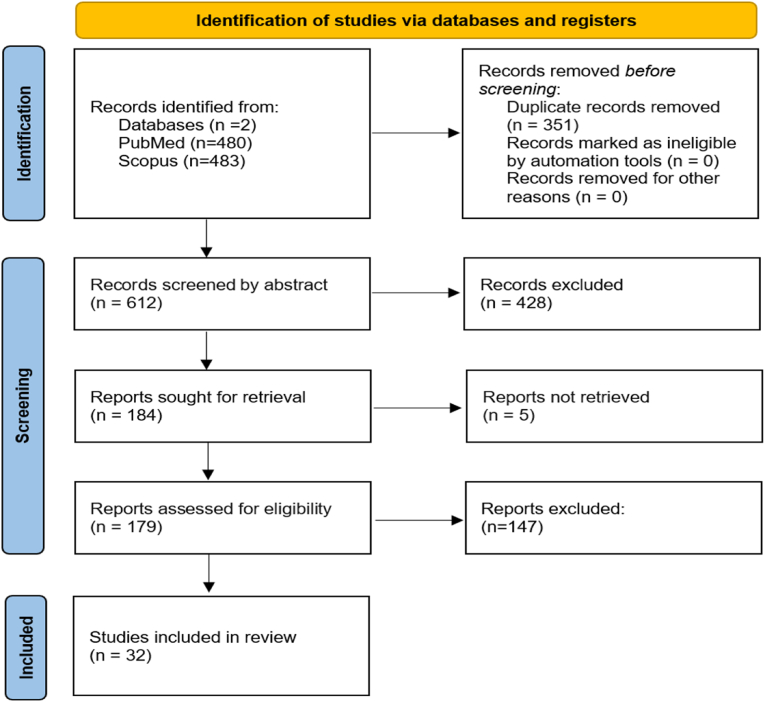
PRISMA flowchart.

The database search yielded a total of 963 studies. After removal of duplicates and abstract screening, 184 articles remained for full-text screening. Out of these, 151 were excluded based on the predefined exclusion criteria. 32 studies were finally included in the review. The PRISMA flowchart is presented in Fig. 1.

**Fig. 2:**
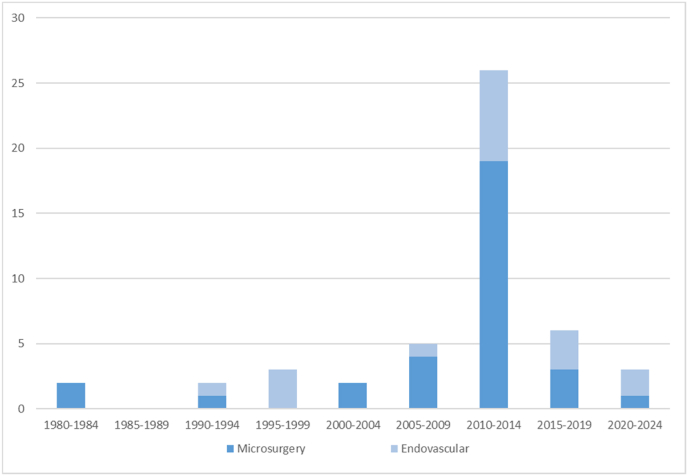
Treated giant aneurysms of the basilar apex reported in the literature by 5-year periods.

The results are summarized in Table 1. There was a total of 49 patients reported in studies fulfilling the inclusion criteria.(See. Fig. 2)

**Table 1:** Giant basilar apex aneurysms treated by open or endovascular techniques. M: male, F: female, SAH: subarachnoid hemorrhage, mRS: modified Rankin Score, BA: basilar artery, C: complete occlusion, I: incomplete occlusion, Mass: mass effect, NA: not available, Endo: endovascular, HH: Hunt-Hess score, VP: ventriculoperitoneal, PCA: posterior cerebral artery, EVD: external ventricular drainage.

Study (Year)	N =	Age (Years)	Sex (M/F)	Size (Max diameter in mm)	Presentation	Previous treatment	Treatment	Complication	Occlusion	preOP mRS	postOP mRS	Follow-up (months)
Rozario and Stein, 1980 (Rozario and Stein, 1980)	1	60	F	26	SAH		Open (BA occlusion)		C	4	1	0.25
Hopkins et al., 1983 (Hopkins et al., 1983)	1	73	M	30	Mass		Open (BA occlusion – bypass)	MidBA occlusion	NA	3	6 (respiratory)	NA
Hirasawa et al., 1992 (Hirasawa et al., 1992)	1	61	F	35	Mass		Endo (balloon occlusion)	Recurrence	I	5	6	2.5
Origitano et al., 1993 (Origitano et al., 1993)	1	58	F	NA	SAH HH 3		Open- clip		NA	4	0	NA
Irie et al., 1997 (Irie et al., 1997)	1	65	F	25	Mass		Endo -Coiling		I	2	0	2
Klein et al., 1997 (Klein et al., 1997)	2	43	M	NA	SAH HH3		Endo -Coiling	PCA occlusion	C	4	2	30
		52	F	NA	SAH HH3		Endo -Coiling		C	4	1	25
Russel et al., 2002 (Russell et al., 2002)	1	45	M	30	Headache	Coiling	Open (BA occlusion)		C	1	1	4
Kim et al., 2002 (Kim et al., 2002)	1	30	M	40	Mass	VP shunt	Open- clip		C	5	1	48
Heppner et al., 2007 (Heppner et al., 2007)	1	48	F	30	Mass		Open- clip	Infarct (MCA -midbrain infarct)	NA	4	6	NA
Takahashi et al., 2007 (Takahashi et al., 2007)	1	29	M	35	Mass	Open (BA occlusion)	Open (PCA occlusion – bypass)		C	3	1	0.5
Iihara et al., 2008 (Iihara et al., 2008)	2	29	M	35	Mass	Open (BA occlusion)	Open (bypass – PCA– PCoA clip)		I	3	1	24.5
		36	F	42	Mass	Endo (BA coil occlusion)	Open (bypass – PCA occlusion)		I	4	1	7.6
Burns et al., 2009 (Burns et al., 2009)	1	44	M	30	SAH		Endo -Coiling	Edema (min. conscious state)	I	3	2	1
Ramanathan et al., 2010 (Ramanathan et al., 2010)	2	72	F	25	Mass	Coil	Open (clip – bypass)		C	2	2	48
		62	F	26	Mass	Coil	Open (BA occlusion – bypass)		I	5	4	18
Miyamoto et al., 2011 (Miyamoto et al., 2011)	4	29	M	35	Mass	Open (BA occlusion)	Open (BA occlusion- Bypass)		C	2	0	24
		37	F	45	Personality disorders	Coil (BA occlusion)	Open (BA occlusion- Bypass)		C	3	0	15
		26	F	30	SAH, Diplopia	Coil	Open (BA occlusion- Bypass)		I	1	0	12
		54	M	28	Diplopia		Open (BA occlusion- Bypass)		C	2	3	9
Derakhsani et al., 2011 (Derakhshani et al., 2011)	1	62	F	25	SAH		Endo -Coiling		I	NA	0	12
Cekirge et al., 2011 (Cekirge et al., 2011)	2	41	F	NA	Mass		Endo -Stent	Recurrence – coiling	I	3	0	24
		45	F	NA	Mass		Endo-Stent		I	3	1	3
Kellner et al., 2011 (Kellner et al., 2011)	2	39	F	30	Mass		Open (BA occlusion)	Midbrain infarct	NA	5	6	NA
		41	M	30	Mass		Open (BA occlusion)	Midbrain infarct	I	4	0	18
Killu and Lanzino 2012 (Killu and Lanzino, 2012)	1	46	F	40	Mass		Open (BA occlusion)		I	3	6	1.5
Park et al., 2012 (Park et al., 2012)	1	58	F	25	SAH		Endo (Stent-coiling)		I	2	0	0.25
Mai et al., 2013 (Mai et al., 2013)	2	47	M	32	Mass		Open (BA occlusion – bypass)		I	2	2	14
		72	F	25	Previous SAH - regrowth	Coil (5 times)	Open (clip- bypass)		C	2	2	48
Nanda et al., 2014 (Nanda et al., 2014a)	7	44	M	NA	NA		Open-clip		NA	1	2	NA
		51	F	NA	NA		Open-clip		NA	1	5	NA
		61	M	NA	NA		Open-clip		NA	1	5	NA
		62	F	NA	NA		Open-clip		NA	1	5	NA
		38	F	NA	NA		Open-clip		NA	1	2	NA
		55	F	NA	NA		Open-clip		NA	1	2	NA
		57	F	NA	NA		Open-clip		NA	5	5	NA
Pyoung Jeon et al., 2014 (Jeon et al., 2014)	2	70	F	26	Recurrence	Coil (2 times)	Endo (Stent-Coiling)	Recurrence (7 months)	C	NA	2	36
		48	M	27	Recurrence - mass	Coil (2 times)	Endo (Stent-coiling)	Recurrence – coiling (5 months)	C	NA	2	12
Shojima et al., 2014 (Shojima et al., 2014)	1	74	F	26	Headache – cognition problems		Open (BA occlusion)	Rupture	NA	2	6	6
Jagadeesan et al., 2014 (Jagadeesan et al., 2014)	1	70+	NA	30	SAH		Endo -Coiling (balloon)	SAH complications	I	NA	6	NA
Samadian et al., 2015 (Samadian et al., 2015)	1	40	M	27	SAH	EVD	Open- clip		C	5	0	0.5
Ge et al., 2016 (Ge et al., 2016)	2	31	F	40	Mass		Endo -Stent-coiling	Mass	I	4	6	0.17
		44	M	25	Mass		Endo -Coiling	Mass	I	3	6	7
Matsukawa et al., 2017 (Matsukawa et al., 2017)	1	67	F	33	NA		Open (BA occlusion - bypass)	Perforator infarct	NA	3	4	12
Signorelli et al., 2017 (Signorelli et al., 2017)	1	76	F	26	Mass	VP shunt	Endo -Stent-coiling	Rerupture	I	5	6	9
Ota et al., 2018 (Ota et al., 2018)	1	67	F	33	NA		Open (Clip -bypass)	Perforator infarct	NA	1	5	NA
Garcia-Perez et al., 2020 (García-Pérez et al., 2020)	1	64	F	25	Mass		Open (BA occlusion)	Recurrence - growth – hydrocephalus – VP shunt	I	3	1	60
Matsuda et al., 2022 (Matsuda et al., 2022)	1	40+	F	32	Mass – infarct	EVD	Endo – Stent- coiling	Recurrence - coiling	I	5	1	6
Killer-Oberpfalzer et al., 2023 (Killer-Oberpfalzer et al., 2023)	1	70+	NA	27	Mass		Endo- Stent-coiling		I	4	4	NA

### 3.1 Epidemiology

**Fig. 3:**
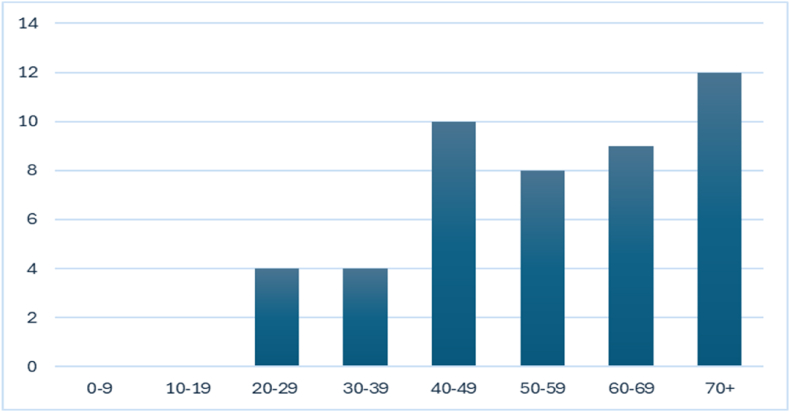
Distribution of the giant aneurysms of the basilar apex according to the patients' age at the time of treatment. Most of the aneurysms were discovered after the 4th decade.

Patients in their 5th to 8th decade of life seem to be more often affected by giant aneurysms of the basilar apex (Fig. 3). (Ujiie et al., 1993) The mean patient age was 51.69 years (SD 14.21). The male-to-female ratio was approximately 1:2, which is comparable to the female preponderance in the literature regarding cerebral aneurysms in general (Cras et al., 2020; Turan et al., 2016). The mean maximum aneurysm reported diameter was 30.57 mm (SD 5.36).(See. Table 2).

**Table 2:** Comparison of clinical and radiological outcome after microsurgical or endovascular treatment. Favorable outcome is defined as mRS 0–2. Worse outcome is defined as a transition from mRS 0–2 to 3–6.

	Endovascular (n = 17)	Open (n = 32)	p-value
Favorable outcome	12/17 (70.6%)	18/32 (56.3%)	0.715
Neurological deterioration	5/15 (33.3%)	14/32 (43.8%)	0.498
Worse Outcome	0/16 (0%)	5/32 (15.6%)	0.159
Death	5/17 (29.4%)	5/32 (15.6%)	0.285
Complete occlusion	4/17 (23.5%)	10/18 (55.6%)	0.053
Recurrence/rerupture	6/17 (35.3%)	2/32 (6.3%)	0.015

### 3.2 Presenting symptoms

Subarachnoid hemorrhage was the presenting symptom in 10/49 (20.4%) of giant basilar apex aneurysms, while 23/49 (46.9%) presented symptoms related to mass effect on the brainstem. 2 patients (4%) presented with headache, 1 (2%) with diplopia, while 1 (2%) only had personality disorders as the presenting symptom. There was 1 case that presented with a cerebellar infarct. In 3 (6.1%) patients, the giant aneurysm was discovered as a recurrence of a previously treated basilar tip aneurysm on follow-up angiography. The patient's presenting condition was not provided in 9 (18.4%) of the cases.

### 3.3 Previous treatment

In 12/49 (24.5%) of the patients, a history of previous treatment of the basilar tip aneurysm was reported. In 7 of those, previous coiling was now followed by open treatment – basilar artery occlusion in 5 and aneurysm clipping combined with bypass in 2. Three patients previously subjected to open basilar artery occlusion were again subjected to microsurgical treatment with trapping of the aneurysm and bypass to the distal PCA. In 2 patients with a history of multiple unsuccessful coiling procedures, further endovascular treatment with Y-stenting of both PCAs and further coiling of the aneurysm was attempted. Of note, both the latter cases presented with a major radiological recurrence, 7 and 5 months postoperatively respectively.

Three patients (6.1%) underwent ventriculoperitoneal shunting or external ventricular drainage before aneurysm treatment due to obstructive hydrocephalus caused by mass effect on the Sylvian aqueduct, while 1 (2%) needed external ventricular drainage due to hydrocephalus caused by SAH.

### 3.4 Treatment modalities

Out of a total 49 patients, 17 (34.7%) received endovascular treatment, while 32 (65.3%) underwent microsurgical treatment. SAH at admission was present in 6 (35.3%) of the patients included in the endovascular treatment group and 4 (12.5%) of the patients in the surgical treatment group (p = 0.06). Endovascular treatment consisted of detachable balloon occlusion in 1 (5.9%), simple coiling in 6 (35.3%), stent-assisted coiling in 7 (41.2%), balloon-assisted coiling in 1 (5.9%) and flow-diverter stenting in 2 (11.8%) of the cases. Microsurgically treated patients were subjected to direct aneurysm clipping in 11 cases (34.4%), clipping combined with revascularization of PCA via bypass in 3 (9.4%), basilar artery occlusion in 7 (21.9%) and basilar artery occlusion combined with bypass to the distal PCA and in select cases occlusion of other vessels of the basilar apex in 11 (34.4%). Microsurgical occlusion of the dominant PCA and/or PCoA as well as the basilar artery was required in 3 (9.4%) of cases.

### 3.5 Radiological and clinical outcomes

Postoperative angiographic result was reported in 17/17 (100%) of the endovascular cases but only 18/32 (56%) of the surgical cases. Out of these, complete occlusion of the aneurysm is reported in 10/18 (55.6%) of the open whereas 4/17 (23.5%) of the endovascular procedures (p < 0.053). Of the latter, only 2 (11.8%), which were amenable to simple coil embolization, were without recurrence at follow-up (Klein et al., 1997).

A favorable clinical outcome (defined as mRS 0–2) was reported on 12/17 (70.6%) of endovascular and 18/32 (56.3%) of the open surgical cases (p = 0.715), although the follow-up duration varied from 5 days to 5 years. Neurological deterioration after treatment was observed in 5/15 (33.3%) of endovascular end 14/32 (43.8%) of open cases (p = 0.498). Five of the thirty-two patients surgically treated (15.6%) experienced worsening outcome (defined as a transition from a mRS 0–2 to 3–6 as compared to their preoperative status), while none of the endovascular patients did (p = 0.159). Death rate was 29.4% for endovascular and 15.6% for open cases (p = 0.285).

Of the surgically treated patients who eventually died, 1 death was unrelated to the procedure and was a result of pulmonary complications, 1 was due to rebleeding and 2 were attributed to postoperative midbrain infarcts. In the endovascular group, 1 death was caused by SAH complications unrelated to the procedure, 1 was a result of rebleeding and 3 were attributed to continued or worsened mass effect of the aneurysm on the brainstem after the operation.

## 4 Discussion

Aneurysm size has been previously reported to correlate with a higher chance of growth and rupture (Greving et al., 2014; Backes et al., 2015). Since giant aneurysms are associated with a rupture risk of up to 50%, treatment is generally warranted if it can be provided with acceptable morbidity (Wiebers, 2003b). Owing to the complexity of such aneurysms, a multimodal endovascular and/or microsurgical treatment is usually attempted. The surgical treatment modalities reported in the cases included in the present study consisted of either simple clipping or clip reconstruction of the aneurysm neck (occasionally with bypass to a sacrificed posterior cerebral artery), or flow reduction – flow alteration techniques. Of the latter, combinations of basilar artery occlusion with occlusion of a dominant PCoA and/or PCA, with or without bypass to the branches of the basilar apex were performed. Endovascular techniques ranged from simple coiling to stent- or balloon-assisted coiling to flow-diverter stenting.

Angiographic complete occlusion rates after treatment were higher after microsurgery (55.6%) compared to endovascular treatment (23.5% - p = 0.053). This trend has been established in findings of large studies on saccular non-giant aneurysms, albeit occlusion rates in non-giant aneurysms and aneurysms in the anterior circulation tend to generally be significantly higher (Wiebers, 2003b; Spetzler et al., 2015). The remarkably high incomplete occlusion rates may signify that a high percentage of treated patients (up to 54.4% of those treated microsurgically and 66.5% of those treated endovascularly) are exposed to aneurysm recurrence/growth or rebleeding. Such a trend was observed in the present review, as a third (6/17 or 35.3%) of the endovascularly treated aneurysms in this review recurred or reruptured. Recurrence in the surgically treated giant basilar tip aneurysms was significantly lower (2/32 or 6.3% - p = 0.015). Endovascular treatment of non-giant saccular aneurysms is generally associated with a higher incomplete occlusion rate and risk of recurrence which is estimated at up to 20% (Wiebers, 2003b; Spetzler et al., 2015; Ferns et al., 2009). Based on the present review, this ratio appears to be even more pronounced in giant aneurysms of the basilar apex, and could be related to the higher incomplete aneurysm occlusion rate after endovascular treatment.

An interesting finding is the fact that of the aneurysms that had previously received additional treatment, the majority were endovascularly treated cases that required microsurgical revision due to recurrence or incomplete occlusion (Russell et al., 2002; Ramanathan et al., 2010; Miyamoto et al., 2011; Mai et al., 2013). This further implies that endovascular treatment is often not a definitive treatment for such lesions.

Major studies that compared treatment modalities for saccular aneurysms have shown a trend towards less morbidity and mortality with endovascular treatment (Wiebers, 2003b; Spetzler et al., 2015). Giant basilar apex aneurysms exhibit a similar morbidity trend, as a favorable outcome (defined as a mRS of 0–2) was achieved in 70.6% of endovascular cases, compared to 56.3% of microsurgical ones. Endovascular treatment was also correlated with lower rates of neurological deterioration (33.3% compared to 43.8% - p = 0.498); no endovascular cases with an initial ‘favorable’ mRS (0–2) progressed to an unfavorable outcome (mRS 3–6)).

However, a notable difference compared to studies on aneurysms of smaller caliber was the significantly higher death rate reported on giant aneurysms treated endovascularly compared with the ones receiving microsurgery. Inspection of the preoperative and postoperative clinical and radiographic details reveals that in most of the endovascular cases, death was a result of continued mass effect on the brainstem after treatment, or rerupture (Hirasawa et al., 1992; Ge et al., 2016). Endovascular embolization in particular seemed to occasionally temporarily aggravate brainstem compression due to edema. This is demonstrated dramatically in the report from Burns et al., where post-embolization transient brainstem edema caused a minimally conscious state, which resolved spontaneously with the resolution of the edema (Burns et al., 2009). These findings could suggest that in cases with significant, clinically apparent brainstem compression preoperatively, due consideration should be given to microsurgical options as a way to ensure maximal brainstem decompression. On the other side, the main cause of neurological deterioration after microsurgical treatment was brainstem infarction due to perforating artery compromise, the avoidance of which is the main technical challenge of this type of surgery.

### 4.1 Limitations

The most important limitation of the present study is selection bias, as it is seldom clear which of the aneurysms were equally amenable to both open and endovascular treatment modalities. Therefore, it is likely that they would either have been selected for the most favorable modality, or for the modality the treating team was most experienced with. Since the available literature includes mostly case series or case reports and the sample size of the patients is relatively small, drawing solid clinical conclusions is precarious at best. Another major limitation is the widely variable follow-up length between the studies, ranging from 5 days to 5 years. Furthermore, it was assumed that in patients whose clinical status was reported as a range of GOS or mRS scores, the mean of the range would correspond to the patients’ status for the purpose of the present study. This may have had the effect of skewing the results toward an unfavorable view of open procedures, as it mainly affected the study by Nanda et al., which is the largest included microsurgical series.

Reports of large series of treated basilar apex aneurysms were also omitted due to not providing explicit data on the imaging or clinical course of individual patients. However, this may skew the analysis by excluding the results of some of the most experienced operators (Lawton, 2002b; Sanai et al., 2008; Nanda et al., 2014b).

Even so, the study provides a useful overview of treatment outcomes of the most popular treatment modalities for giant basilar apex aneurysms and can serve as a reference frame for clinical decision-making.

## 5 Conclusion

Particularities related to aneurysm morphology and afferent/efferent vessel anatomy influence the selection of treatment technique. This is particularly important for giant aneurysms of the basilar apex, where no perfect treatment modality seems to exist and a delicate balance needs to be maintained between complete lesion obliteration and patient safety. Due to the small sample size, as well as lesion and treatment modality heterogeneity in the available literature, solid conclusions regarding choice of treatment cannot be drawn. Microsurgical treatment of giant saccular aneurysms of the basilar apex appears to be associated with increased morbidity but also higher rates of complete occlusion and lower recurrence rates than endovascular treatment. In cases with severe brainstem compression, microsurgical treatment may present a better chance of mass effect resolution, which may translate to lower mortality. However, every giant basilar apex aneurysm has its own particular challenges, therefore decision-making should be individualized on a case-by-case basis, ideally in an interdisciplinary setting with expertise in both endovascular surgery and microsurgery.

## Declaration of competing interest

The authors declare that they have no known competing financial interests or personal relationships that could have appeared to influence the work reported in this paper.

## References

[bib1] Backes D., Vergouwen M.D.I., Tiel Groenestege A.T. (2015). PHASES score for prediction of intracranial aneurysm growth. Stroke.

[bib2] Burns J.D., Rabinstein A.A., Cloft H., Lanzino G., Daniels D.J., Wijdicks E.F.M. (2009). Minimally conscious state after ruptured giant basilar aneurysm. Arch. Neurol..

[bib3] Cekirge H.S., Yavuz K., Geyik S., Saatci I. (2011). A novel “Y” stent flow diversion technique for the endovascular treatment of bifurcation aneurysms without endosaccular coiling. Am. J. Neuroradiol..

[bib4] Choi I.S., David C. (2003). Giant intracranial aneurysms: development, clinical presentation and treatment. Eur. J. Radiol..

[bib5] Cras T.Y., Bos D., Ikram M.A. (2020). Determinants of the presence and size of intracranial aneurysms in the general population. Stroke.

[bib6] Da Ros V., Caroff J., Rouchaud A. (2017). Large basilar apex aneurysms treated with flow-diverter stents. Am. J. Neuroradiol..

[bib7] Derakhshani S., Rosa S., Kleidona I.A., Haliasos N., Chawda S. (2011). Embolization of a wide neck giant basilar tip aneurysm using two coils. NeuroRadiol. J..

[bib8] Ferns S.P., Sprengers M.E.S., van Rooij W.J. (2009). Coiling of intracranial aneurysms. Stroke.

[bib9] Gaastra B., Ren D., Alexander S. (2022). Evidence-based interconversion of the Glasgow Outcome and modified Rankin scales: pitfalls and best practices. J. Stroke Cerebrovasc. Dis..

[bib10] García-Pérez D., Panero I., Eiriz C. (2020). Delayed extensive brain edema caused by the growth of a giant basilar apex aneurysm treated with basilar artery obliteration: a case report. BMC Neurol..

[bib11] Ge H., Li Y., Lv X. (2016). A challenging entity of unruptured giant saccular aneurysms of vertebrobasilar artery. Neurol. Neurochir. Pol..

[bib12] Ge H., Chen X., Liu K. (2022). Endovascular treatment of large or giant basilar artery aneurysms using the pipeline embolization device: complications and outcomes. Front. Neurol..

[bib13] Greving J.P., Wermer M.J.H., Brown R.D. (2014). Development of the PHASES score for prediction of risk of rupture of intracranial aneurysms: a pooled analysis of six prospective cohort studies. Lancet Neurol..

[bib14] Heppner P.A., Ellegala D.B., Robertson N., Nemergut E., Jaganathan J., Mee E. (2007). Basilar tip aneurysm – adenosine induced asystole for the treatment of a basilar tip aneurysm following failure of temporary clipping. Acta Neurochir..

[bib15] Hirasawa T., Tsubokawa T., Katayama Y. (1992). Growth of a giant aneurysm following complete thrombosis by detachable balloon occlusion. Surg. Neurol..

[bib16] Hopkins L.N., Budny J.L., Castellani D. (1983). Extracranial-intracranial arterial bypass and basilar artery ligation in the treatment of giant basilar artery aneurysms. Neurosurgery.

[bib17] Iihara K., Murao K., Yamada N. (2008). Growth potential and response to multimodality treatment of partially thrombosed large or giant aneurysms in the posterior circulation. Neurosurgery.

[bib18] Irie K., Taki W., Nakahara I., Sakai N., Isaka F., Kikuchi H. (1997). Endovascular treatment of a partially thrombosed giant basilar tip aneurysm using interlocking detachable coils —case report. Neurol. Med.-Chir..

[bib19] Jagadeesan B.D., Siddiq F., Grande A.W., Tummala R.P. (2014). Modified balloon assisted coil embolization for the treatment of intracranial and cervical arterial aneurysms using coaxial dual lumen balloon microcatheters: initial experience. J Neurointerv Surg.

[bib20] Jeon P., Kim B.M., Kim D.J., Kim DI k, Park K.Y. (2014). Y-configuration double-stent-assisted coiling using two closed-cell stents for wide-neck basilar tip aneurysms. Acta Neurochir..

[bib21] Kellner C.P., Haque R.M., Meyers P.M., Lavine S.D., Connolly E.S., Solomon R.A. (2011). Complex basilar artery aneurysms treated using surgical basilar occlusion: a modern case series. J. Neurosurg..

[bib22] Killer-Oberpfalzer M., Chapot R., Orion D., Barr J.D., Cabiri O., Berenstein A. (2023). Clinical experience with the Bendit steerable microcatheter: a new paradigm for endovascular treatment. J Neurointerv Surg.

[bib23] Killu A.M., Lanzino G. (2012). Giant basilar-artery aneurysm. N. Engl. J. Med..

[bib24] Kim M.S., Oh C.W., Han D.H. (2002). Growth of basilar artery aneurysm after ventriculo-peritoneal shunt. J. Clin. Neurosci..

[bib25] Klein G.E., Szolar D.H., Leber K.A., Karaic R., Hausegger K.A. (1997). Basilar tip aneurysm: endovascular treatment with Guglielmi detachable coils-midterm results. Radiology.

[bib26] Lawton M.T. (2002). Basilar apex aneurysms: surgical results and perspectives from an initial experience. Neurosurgery.

[bib27] Lawton M.T. (2002). Basilar apex aneurysms: surgical results and perspectives from an initial experience. Neurosurgery.

[bib28] Locksley H.B. (1966). Section V, part I: natural history of subarachnoid hemorrhage, intracranial aneurysms and arteriovenous malformations. J. Neurosurg..

[bib29] Mai J.C., Tariq F., Kim L.J., Sekhar L.N. (2013). Flow diversion radial artery bypass graft coupled with terminal basilar artery occlusion for the treatment of complex basilar apex aneurysms. Operative Neurosurgery.

[bib30] Matsuda Y., Terada T., Tetsuo Y., Tsumoto T. (2022). Partially thrombosed giant basilar tip aneurysm that remarkably decreased in size after stent-assisted coiling associated with the disappearance of neovascularization. Neuroradiology.

[bib31] Matsukawa H., Kamiyama H., Miyazaki T. (2017). Surgical treatment of unruptured distal basilar artery aneurysm: durability and risk factors for neurological worsening. Acta Neurochir..

[bib32] Miyamoto S., Funaki T., Iihara K., Takahashi J.C. (2011). Successful obliteration and shrinkage of giant partially thrombosed basilar artery aneurysms through a tailored flow reduction strategy with bypass surgery. J. Neurosurg..

[bib33] Nanda A., Sonig A., Banerjee A.D., Javalkar V.K. (2014). Microsurgical management of basilar artery apex aneurysms: a single surgeon's experience from Louisiana state university, shreveport. World Neurosurg.

[bib34] Nanda A., Sonig A., Banerjee A.D., Javalkar V.K. (2014). Microsurgical management of basilar artery apex aneurysms: a single surgeon's experience from Louisiana state university, shreveport. World Neurosurg.

[bib35] Origitano T.C., Anderson D.E., Tarassoli Y., Howard Reichman O., Al-Mefty O. (1993). Skull base approaches to complex cerebral aneurysms. Surg. Neurol..

[bib36] Ota N., Matsukawa H., Noda K. (2018). Evaluation of microsurgery for managing giant or complex cerebral aneurysms: a retrospective study. World Neurosurg.

[bib37] Park H.R., Yoon S.M., Shim J.J., Kim S.H. (2012). Waffle-cone technique using solitaire AB stent. J Korean Neurosurg Soc.

[bib38] Ramanathan D., Ciporen J., Ghodke B., Ellenbogen R.G., Sekhar L.N. (2010). Treatment of coil embolization failed recurrent giant basilar tip aneurysms with bypass and surgical occlusion. J Neurointerv Surg.

[bib39] Rozario R.A., Stein B.M. (1980). Ligation of the basilar artery as the definitive treatment for a giant aneurysm of the basilar artery apex. Neurosurgery.

[bib40] Russell S.M., Nelson P.K., Jafar J.J. (2002). Neurological deterioration after coil embolization of a giant basilar apex aneurysm with resolution following parent artery clip ligation. J. Neurosurg..

[bib41] Samadian M., Alavi E., Bakhtevari M.H., Rezaei O. (2015). Surgical management of giant basilar tip aneurysm associated with moyamoya disease: a case report and literature review. World Neurosurg.

[bib42] Sanai N., Tarapore P., Lee A.C., Lawton M.T. (2008). The current role of microsurgery for posterior circulation aneurysms. Neurosurgery.

[bib43] Shojima M., Morita A., Kimura T., Oshima M., Kin T., Saito N. (2014). Computational fluid dynamic simulation of a giant basilar tip aneurysm with eventual rupture after hunterian ligation. World Neurosurg.

[bib44] Signorelli F., Sturiale C.L., La Rocca G. (2017). Neck Reconstruction with pCONus-Assisted Coiling.

[bib45] Spetzler R.F., McDougall C.G., Zabramski J.M. (2015). The barrow ruptured aneurysm trial: 6-year results. J. Neurosurg..

[bib46] Takahashi J.C., Murao K., Iihara K. (2007). Successful “blind-alley” formation with bypass surgery for a partially thrombosed giant basilar artery tip aneurysm refractory to upper basilar artery obliteration. J. Neurosurg..

[bib47] Tjahjadi M., Serrone J., Hernesniemi J. (2018). Should we still consider clips for basilar apex aneurysms? A critical appraisal of the literature. Surg. Neurol. Int..

[bib48] Turan N., Heider R.A.J., Zaharieva D., Ahmad F.U., Barrow D.L., Pradilla G. (2016). Sex differences in the formation of intracranial aneurysms and incidence and outcome of subarachnoid hemorrhage: review of experimental and human studies. Transl Stroke Res.

[bib49] Ujiie H., Sato K., Onda H. (1993). Clinical analysis of incidentally discovered unruptured aneurysms. Stroke.

[bib50] van Rooij W.J., Sluzewski M. (2007). Coiling of very large and giant basilar tip aneurysms: midterm clinical and angiographic results. Am. J. Neuroradiol..

[bib51] van Rooij W.J., Sluzewski M. (2009). Endovascular treatment of large and giant aneurysms. Am. J. Neuroradiol..

[bib52] Vlak M.H., Algra A., Brandenburg R., Rinkel G.J. (2011). Prevalence of unruptured intracranial aneurysms, with emphasis on sex, age, comorbidity, country, and time period: a systematic review and meta-analysis. Lancet Neurol..

[bib53] Wiebers D.O. (2003). Unruptured intracranial aneurysms: natural history, clinical outcome, and risks of surgical and endovascular treatment. Lancet.

[bib54] Wiebers D.O. (2003). Unruptured intracranial aneurysms: natural history, clinical outcome, and risks of surgical and endovascular treatment. Lancet.

